# Alzheimer's Disease Amyloid-β Links Lens and Brain Pathology in Down Syndrome

**DOI:** 10.1371/journal.pone.0010659

**Published:** 2010-05-20

**Authors:** Juliet A. Moncaster, Roberto Pineda, Robert D. Moir, Suqian Lu, Mark A. Burton, Joy G. Ghosh, Maria Ericsson, Stephanie J. Soscia, Anca Mocofanescu, Rebecca D. Folkerth, Richard M. Robb, Jer R. Kuszak, John I. Clark, Rudolph E. Tanzi, David G. Hunter, Lee E. Goldstein

**Affiliations:** 1 Molecular Aging & Development Laboratory, Department of Surgery, Brigham and Women's Hospital, Harvard Medical School, Boston, Massachusetts, United States of America; 2 Department of Ophthalmology, Massachusetts Eye and Ear Infirmary, Harvard Medical School, Boston, Massachusetts, United States of America; 3 Genetics and Aging Research Unit, Department of Neurology, Massachusetts General Hospital, Harvard Medical School, Charlestown, Massachusetts, United States of America; 4 Electron Microscopy Facility, Department of Cell Biology, Harvard Medical School, Boston, Massachusetts, United States of America; 5 Department of Pathology, Brigham and Women's Hospital, Harvard Medical School, Boston, Massachusetts, United States of America; 6 Department of Ophthalmology, Children's Hospital Boston, Harvard Medical School, Boston, Massachusetts, United States of America; 7 Departments of Ophthalmology and Pathology, Rush University Medical Center, Chicago, Illinois, United States of America; 8 Departments of Biological Structure and Ophthalmology, University of Washington, Seattle, Washington, United States of America; Mayo Clinic, United States of America

## Abstract

Down syndrome (DS, trisomy 21) is the most common chromosomal disorder and the leading genetic cause of intellectual disability in humans. In DS, triplication of chromosome 21 invariably includes the *APP* gene (21q21) encoding the Alzheimer's disease (AD) amyloid precursor protein (APP). Triplication of the *APP* gene accelerates APP expression leading to cerebral accumulation of APP-derived amyloid-β peptides (Aβ), early-onset AD neuropathology, and age-dependent cognitive sequelae. The DS phenotype complex also includes distinctive early-onset cerulean cataracts of unknown etiology. Previously, we reported increased Aβ accumulation, co-localizing amyloid pathology, and disease-linked supranuclear cataracts in the ocular lenses of subjects with AD. Here, we investigate the hypothesis that related AD-linked Aβ pathology underlies the distinctive lens phenotype associated with DS. Ophthalmological examinations of DS subjects were correlated with phenotypic, histochemical, and biochemical analyses of lenses obtained from DS, AD, and normal control subjects. Evaluation of DS lenses revealed a characteristic pattern of supranuclear opacification accompanied by accelerated supranuclear Aβ accumulation, co-localizing amyloid pathology, and fiber cell cytoplasmic Aβ aggregates (∼5 to 50 nm) identical to the lens pathology identified in AD. Peptide sequencing, immunoblot analysis, and ELISA confirmed the identity and increased accumulation of Aβ in DS lenses. Incubation of synthetic Aβ with human lens protein promoted protein aggregation, amyloid formation, and light scattering that recapitulated the molecular pathology and clinical features observed in DS lenses. These results establish the genetic etiology of the distinctive lens phenotype in DS and identify the molecular origin and pathogenic mechanism by which lens pathology is expressed in this common chromosomal disorder. Moreover, these findings confirm increased Aβ accumulation as a key pathogenic determinant linking lens and brain pathology in both DS and AD.

## Introduction

Down syndrome (DS, [1]) is the leading genetic cause of intellectual disability and the most common chromosomal disorder compatible with human survival [2], [3], [4], [5]. DS affects approximately 1 in 700 live births and an estimated 220,000 newborns each year [6]. In the majority of cases, the disorder arises from sporadic non-disjunction of chromosome 21 (HSA21) and triplication of the entire chromosome [7], [8], [9], or infrequently, from partial aneuploidy due to unbalanced chromosomal translocation [10], [11], [12], [13]. In most cases, DS aneuploidy disrupts gene-dose equilibrium in all somatic cells, whereas a minority of cases demonstrates mosaicism [14]. Chromosomal triplication invariably includes the *APP* gene (21q21) that encodes the amyloid precursor protein, APP [15], [16]. Endoproteolytic cleavage of APP yields the pathogenic amyloid-β peptides (Aβ) that progressively accumulate in the brain as the diffuse and neuritic plaques of Alzheimer's disease (AD). In DS, cerebral Aβ accumulation is greatly accelerated [17] and leads to invariant early-onset AD neuropathology and age-dependent neurocognitive sequelae [18], [19].

Lens abnormalities in subjects with clinical features of DS were first reported over a century ago [20], [21] followed by numerous confirmatory reports since [20], [22], [23], [24]. The distinctive DS lens phenotype clinically manifests as cerulean “blue dot” opacities that often emerge in the first decade of life, and in some cases, may be evident at birth [22], [25], [26], [27]. DS cataracts typically localize to the supranuclear region and are histologically characterized by granular material of uncertain composition [20], [24], [25]. The molecular origin and pathogenic mechanisms by which the DS aneuploidy is expressed as a distinctive age-dependent lens phenotype are unknown.

We previously reported disease-linked supranuclear cataracts that correlate with pathogenic Aβ accumulation, classical amyloid pathology, and co-localizing pathology in lenses obtained from subjects with Alzheimer's disease (AD) but not in those with other neurodegenerative disorders nor in normal aged controls [28]. In AD lenses, Aβ accumulates as electron-dense cytosolic aggregates (longest axial dimension, ∼5 nm to ∼200 nm) that distribute heterogeneously within the cytoplasm of supranuclear and deep cortical lens fiber cells. AD-linked Aβ aggregates in the lens qualify as Raleigh scattering centers that clinically manifest as supranuclear opacities that ultimately progress to frank cataracts. These AD-linked supranuclear cataracts are phenotypically, anatomically, ultrastructurally, and biochemically distinguishable from common age-related nuclear cataracts. Given the association between Aβ amyloid lens pathology in AD [28], we hypothesized that subjects with DS would also demonstrate age-related Aβ amyloid pathology in the lens. The aim of the present study was to test the hypothesis that DS-linked lens pathology reflects accelerated Aβ accumulation and co-localizing amyloid pathology that clinically manifests as the characteristic supranuclear cataract phenotype associated with this common chromosomal disorder.

## Methods

### Subjects and Specimens

Lens specimens were obtained from the following sources: (i) subjects with DS requiring cataract surgery (n = 3 males: 36, 46, and 47 years of age) at the Massachusetts Eye and Ear Infirmary; (ii) postmortem specimens from donors with DS (n = 12 total; n = 9 males: 2 to 69 years of age; n = 3 females: 42, 47, and 61 years of age) and normal controls (n = 34 total; n = 20 males: 7 months to 88 years of age; n = 14 females: 2 to 82 years of age) procured through national tissue networks (National Disease Registry Interchange, Philadelphia; PA; Florida Lion's Eye Bank, Miami, FL; Sun Health Research Institute, Sun City, AZ); and (iii) archival lens specimens generously provided by Dr. Richard Robb, Children's Hospital, Harvard Medical School, Boston, MA (DS, n = 4 total; n = 3 males: 1 day, 3 weeks, and 22 years of age; n = 1 female: 7 months of age. Normal controls, n = 3 total; n = 2 males, 1 day and 21 years of age; n = 2 females, 2 days and 4 months of age). An earlier histochemical analysis of lenses from this archival collection was reported in 1978 [24]. Postmortem brain specimens (superior temporal cortex) were obtained from the Massachusetts Alzheimer's Disease Research Center Brain Bank (Massachusetts General Hospital, Boston, MA, USA), and included the following donor groups: AD (CERAD criteria) n = 3 total; n = 1 male: 86 years of age; n = 2 females: 76 and 81 years of age); DS (karyotype confirmed) n = 4 total; n = 4 males: 56, 57, 58, and 60 years of age); normal controls (n = 3 total; n = 1 male: 86 years of age; n = 2 females: 72 and 88 years of age). Clinical information regarding cytogenetic microanalysis and chromosomal subtyping was not available. Lenses obtained from intact postmortem eyes were the only specimens used for biochemical analysis. All lenses obtained from intact, non-traumatic globes were included in the study and assigned to cohorts based on available clinical information. No other selection criteria were applied.

Ethical review and permission to conduct this investigation were approved by Harvard Medical School, Brigham and Women's Hospital, Massachusetts Eye and Ear Infirmary, and Children's Hospital Boston. This study conforms to applicable regulatory guidelines at Harvard Medical School and principles of human research subject protection in the Declaration of Helsinki. Patients were informed, consent forms signed, and patient samples collected and signed in accordance with an approved IRB protocol (02-08-053X) at the Massachusetts Eye and Ear Infirmary, Boston, MA, USA.

### Slit Lamp Biomicroscopy

Clinical ophthalmological examinations included slit lamp stereophotomicroscopy and microvideography following routine pupillary dilation. In three DS cases (n = 3 males: 36, 46, and 47 years of age), slit lamp photomicrographs were obtained in conjunction with cataract surgery. Patients were informed and signed consent forms in accordance with an approved IRB protocol at the Massachusetts Eye and Ear Infirmary, Boston, MA, USA. Stereophotomicroscopic analysis of dissected intact human lenses was performed on postmortem specimens obtained from enucleated eyes procured through national tissue bank resources (National Disease Registry Interchange, Philadelphia; PA; Florida Lion's Eye Bank, Miami, FL). Harvested lenses were bathed in buffered artificial aqueous humor (295 mOsm, pH 7.20) at 37°C, visualized with a custom-adapted surgical slit lamp stereophotomicroscope (Carl Zeiss, Thornwood, NY) equipped with a stereo beamsplitter (Urban Engineering, Burbank, CA) and side-arm digital camera assembly (Nikon, Melville, NY).

### Histopathology, Immunohistochemistry, Light and Electron Microscopy

A portion of the present study included re-analysis of lens specimens from an archival collection generously provided by co-author Richard Robb, M.D., Department of Ophthalmology, Children's Hospital Boston, Harvard Medical School, Boston, MA. Paraffin-embedded specimens in this collection date back to the 1960's and have been previously described [24]. Original paraffin blocks in this archival collection were highly friable and required re-embedding in fresh paraffin prior to histological processing. For all histological studies, routine tissue sections (8 µm) were obtained by microtomy, immunostained with Aβ monoclonal antibody 6E10 directed against Aβ_3–8_ or 4G8 directed against Aβ_18–22_ (generously provided by Joseph Bertelsen, Signet Laboratories, Covance, Dedham, MA), and processed by conventional immunohistochemistry using a commercial kit (Vector Laboratories, Burlingame, CA) according to the manufacturer's instructions. Bennhold's modified Congo red staining method [29] was used for amyloid histopathology. Brightfield and cross-polarization photomicroscopy were conducted using a Nikon Eclipse E600 upright microscope (Nikon USA, Melville, NY) equipped with a Spot Slider RT digital CCD photomicroscope camera and proprietary image acquisition software (Diagnostic Instruments, Sterling Heights, MI).

### Immunogold Electron Microscopy

Immunogold electron microscopy (IEM) was conducted as previously described using ultra-thin cryosections without plastic embedding [28]. For IEM analyses, specimens were adsorbed to a carbon-coated grid, blocked for 10 min in 1% BSA, immunolabeled with the first detection antibody using anti-Aβ monoclonal antibody 4G8 (Signet Laboratories, Covance, Dedham, MA), then incubated with rabbit anti-mouse bridging antibody followed by incubation with 10 nm immunogold-conjugated Protein A (Jan Slot, Utrecht, Netherlands). Grids were fixed briefly in 1% glutaraldehyde, floated on 4 drops of 0.02M glycine/PBS to quench free aldehyde groups, then immunolabeled with the second detection antibody using a rabbit anti-αB-crystallin polyclonal antibody (Dr. Jack Liang, Brigham & Women's Hospital, Boston, MA, USA) or normal rabbit serum control. Specimens were then exposed to 15 nm immunogold-conjugated Protein A (Jan Slot, Utrecht, Netherlands), negatively stained with uranyl acetate, and examined on a JEOL 1200EX transmission electron microscope at the Electron Microscope Facility, Harvard Medical School. All immunohistochemical analyses included specificity controls in which identically processed specimens were incubated with detection antibody that was immunodepleted by pre-absorption with synthetic antigen (human Aβ42, Keck Laboratory, Yale University, New Haven, CT) as previously described[28].

### Tryptic Digest Tandem Mass Spectrometry Protein Sequencing

Lens specimens were homogenized in 70% formic acid, ultra-centrifuged at 100,000×*g* for 1 h at 4°C, and fractions separately retained for biochemical analysis. Supernatant was concentrated by nitrogen stream, neutralized with NaOH, and diluted 50-fold in TBS containing 10 µg anti-Aβ monoclonal antibody 6E10 (Signet Laboratories, Covance, Dedham, MA). Immunocomplexes were precipitated with magnetic beads covalently coupled to sheep anti-mouse antibody (Invitrogen, Carlsbad, CA), separated from unbound material by exposure to a magnetic field, and released by acidification in 100 mM glycine at pH 3. Samples were then subjected to reverse phase HPLC on a ZORBAX SB-C8 column (Agilent, Santa Clara, CA) and eluted under isocratic conditions (0.1% TFA; 30.5% acetonitrile) at 1 ml/min at 80°C. Elution times were determined by comparison to chromatographic profiles of synthetic human Aβ (Keck Laboratory, Yale University, New Haven, CT). Isolated fractions were dried by vacuum centrifugation, digested with trypsin, and fractionated by reverse phase HPLC. Post-digest eluates were subjected to electrospray ionization and ESI-tandem MS/MS LCQ-DECA ion-trap mass spectrometry (ThermoFinnigan, San Jose, CA) at the Taplin Mass Spectrometry Facility, Harvard Medical School. Peptide sequences were identified by computer-assisted search program (Sequest, Thermo Fisher Scientific, Waltham, MA).

### Aβ Fluoro-ELISA and Aβ Immunoblotting

Analytical quantification of human Aβ40 and human Aβ42 was accomplished using two commercially available human Aβ specific ELISA kits (BetaMark, Covance, Dedham, MA; BioSource, Invitrogen, San Diego, CA). For experiments utilizing SDS extracted tissue, we used a commercially available human Aβ ELISA kit (BetaMark, Covance, Dedham, MA) that is resistant to interference from detergent. This ELISA assay system is compatible with tissue extracts containing SDS with minimal loss in sensitivity. Samples containing SDS were diluted to a final detergent concentration of less than 0.1%. For experiments utilizing formic acid extracted tissue, we used a second commercially available Aβ ELISA kit (BioSource, Invitrogen, San Diego, CA). For immunoblotting analysis, lens subcellular fractions were normalized for protein concentration and analyzed as previously described [28]. Blots were probed with human Aβ-specific monoclonal antibody WO2 (Genetics Company, Zurich, Switzerland) directed against human Aβ_4–10_ or monoclonal antibody 6E10 (Signet Laboratories, Covance, Dedham, MA) directed against human Aβ_3–8_.

### Quasi-Elastic Light Scattering Analysis of Human Lens Protein Suspensions

Quasi-elastic light scattering (QLS) experiments were conducted using a custom laboratory instrument (Neuroptix Corporation, Acton, MA) that delivers linearly polarized continuous wave near-infrared laser light (λ = 780 nm; focused beam diameter, 30–35 µm; power output = 0.3 mW). The instrument incorporates a rigidly positioned pinhole mirror (aperture diameter = 100 µm) for selective detection of backscattered photons at a fixed scattering angle (θ = 120 degrees). Signal detection and analysis of backscattered light intensity was accomplished with an avalanche photodiode, integrated 256-channel multi-tau digital autocorrelator (τ = 480 ns), and custom software analysis package. Analyses involving aqueous suspensions of water-soluble lens proteins were conducted in cylindrical optical glass specimen vials positioned in a rigid holder mounted to the instrument. Three series of five independent measurements (each 1.3 seconds in duration) were performed for a total of fifteen measurements comprising each averaged test point. To investigate the interaction of Aβ peptides with other cytosolic lens proteins, a membrane-free water-soluble protein extract was prepared from freshly dissected human lenses obtained from postmortem donor eyes (National Disease Registry Interchange, Philadelphia; PA; Florida Lion's Eye Bank, Miami, FL). Dissected lenses were mechanically homogenized, ultracentrifuged at 100,000×g for 1 h at 4°C, and diluted to 1 mg/ml in phosphate buffered saline, pH 7.20. This aqueous lens protein extract is predominantly composed of water-soluble proteins in the cytoplasm of lens fiber cells. Purified synthetic human Aβ42 or Aβ40 (Keck Laboratories, Yale University, New Haven, CT) was added to freshly prepared human lens protein extract and incubated in siliconized Eppendorf tubes in a humidified 37°C 5% CO_2_-balanced tissue culture chamber prior to light scattering analysis. All samples were incubated in the dark to reduce photodynamic effects. For microscopic visualization, aliquots of the incubated samples were placed on a microscope slide as wet-mount preparations and visualized by conventional photomicroscopy under brightfield and cross-polarized illumination. Immunogold electron microscopic analysis of incubated material was conducted as detailed above.

## Results

### Supranuclear Cataract Pathology in Down Syndrome Lenses

**Figure 1:**
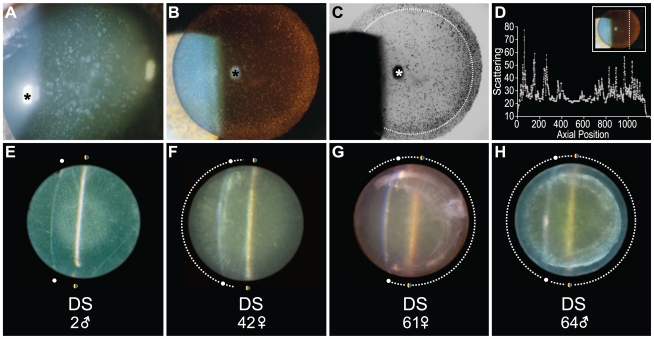
Phenotypic expression of representative supranuclear cataracts in Down syndrome. (**A-C**) Representative supranuclear cataract in a 46-year-old male with Down syndrome observed by slit lamp biomicroscopy. (**A**) Broad-beam illumination demonstrates numerous cerulean coronary “blue dot” lens opacities. Asterisk denotes first Purkinje image (corneal specular reflection). (**B**) Retro-illumination reveals a distinctive peripheral ring of opacification in the subequatorial supranuclear subregion of the lens. Red reflex is imparted by retinal reflection. Corneal Purkinje image is evident centrally (asterisk). (**C**) Inverted grayscale rendering of the retro-illumination image highlights the circumferential subequatorial supranuclear opacification (dashed circle) that characterizes the distinctive Down syndrome cataract phenotype. Corneal Purkinje image is evident centrally (asterisk). (**D**) Static light scattering intensity plotted as a function of anatomical position within the lens. Axial location is referenced to the dashed line and anatomical orientation axes represented in the inset. (**E-H**) Representative stereophotomicroscopic images of *ex vivo* lenses from subjects with Down syndrome of indicated ages and gender. Colored dots indicate superior and inferior extent of the slit beam ribbon defining the posterior surface of each imaged lens. White dots indicate superior and inferior extent of the light reflex on the anterior aspect of each imaged lens. (**E**) Lens from a 2-year-old male with Down syndrome. Slit beam photomicroscopy demonstrates a grossly normal transparent lens. (**F**) Lens from a 42-year-old female with Down syndrome. Supranuclear opacification is evident in a semicircular arc (dashes) with prominent cortical spokes and moderate lenticular brunescence. (**G**) Lens from a 61-year-old female with Down syndrome. Distinctive supranuclear opacification describes an incomplete supranuclear arc (dashes). Cortical spokes and moderate lenticular brunescence are present. (**H**) Lens from a 64-year-old male with Down syndrome. Prominent circumferential opacification is present as a subequatorial supranuclear ring cataract (dashed circle). This cataract describes an annular half torroid that follows the anteroposterior orientation of the supranuclear fiber cells enveloping the peripheral extent of the embryonic nucleus. Cortical spokes and moderate lenticular brunescence are evident. This same lens is presented as a stereo image pair with and without intact zonule fibers (Fig. S1 and Fig. 2, respectively). See text for details.

Slit lamp examination of subjects with karyotype-confirmed DS revealed classical cerulean “blue dot” cataracts (Fig 1*A*) with a characteristic subequatorial supranuclear distribution (Fig. 1*B,C*) and corresponding increased localized static light scattering (Fig. 1*D*). The anatomical localization of the diffuse opacities observed by indirect ophthalmoscopy (Fig. 1*A*) correspond to a distinctly annular supranuclear distribution as revealed by retroillumination (Fig. 1*B,C*) and confirmed by scattering densitometry (Fig. 1*D*) of the same lens. The lens from a 2-year-old male with DS demonstrated grossly normal transparency without evidence of clinically detectable lenticular pathology (Fig. 1*E*). By contrast, the DS supranuclear cataract phenotype was identified in all adult DS patients clinically examined and in all postmortem lens specimens from adult DS donors. Lenses from mature DS subjects demonstrated apparent age-dependent supranuclear lens pathology (Fig. *1E-H*) that was phenotypically indistinguishable from that previously reported in late-onset sporadic AD (Fig. 1*F-H*) [*28*].

**Figure 2:**
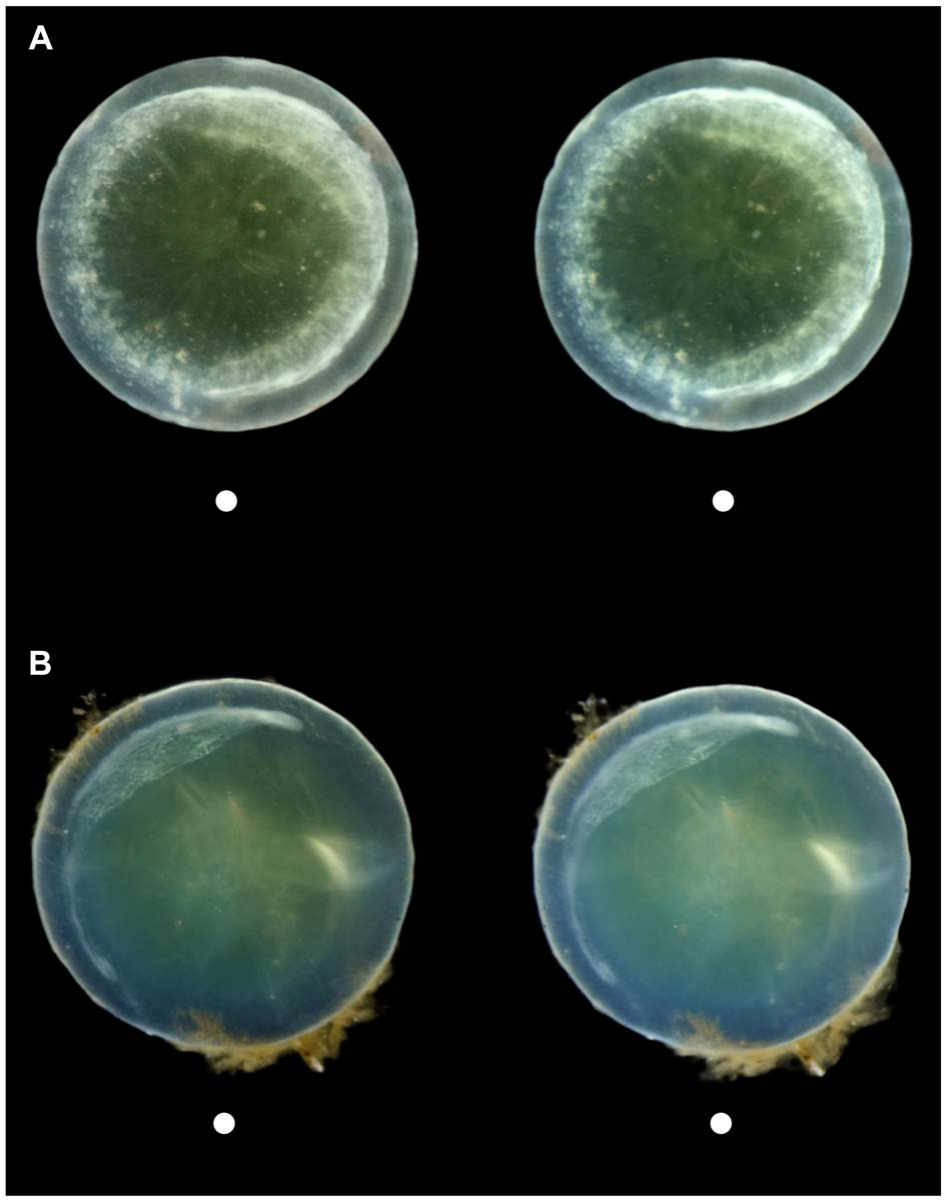
Stereo images demonstrating mature supranuclear lens pathology in Down syndrome and Alzheimer's Disease. (**A**) Characteristic circumferential supranuclear cataract in the lens of a 64-year-old male subject with Down syndrome. This distinctive cataract is evident as an annular half-toroid band of opacification in the deep cortical and supranuclear subregions of the lens. This same lens specimen is presented as a slit lamp biomicrocopic image (Fig. 1H) and as a stereo image pair (with intact zonule fibers, Fig. S1). This dramatic Down syndrome cataract is phenotypically comparable to the incomplete subequatorial supranuclear cataract observed in the lens of a 76-year-old male subject with advanced Alzheimer's disease (**B**). These distinctive supranuclear cataracts are not observed in age-normal control subjects. See text for details.

A representative stereo image of a lens from a 64-year-old male with DS (Fig. 2*A*) illustrates an advanced circumferential supranuclear cataract characteristic of the DS phenotype. The phenotypic features of this distinctive cataract phenotype are demonstrated in the same lens observed by slit lamp photomicroscopy (Fig. 1*H*) and as a stereo image pair with intact zonule fibers (Fig. S1). The cataract phenotype observed in DS was strikingly similar to the mature subequatorial supranuclear cataracts previously reported in advanced AD (Fig. *2B* *;* [28]). This distinctive DS-associated cataract was not present in age-matched normal control subjects nor was this focal lens pathology associated with global swelling, diffuse opacification, or capsular disruption indicative of postmortem tissue injury.

### Histochemical and Ultrastructural Analyses

**Figure 3:**
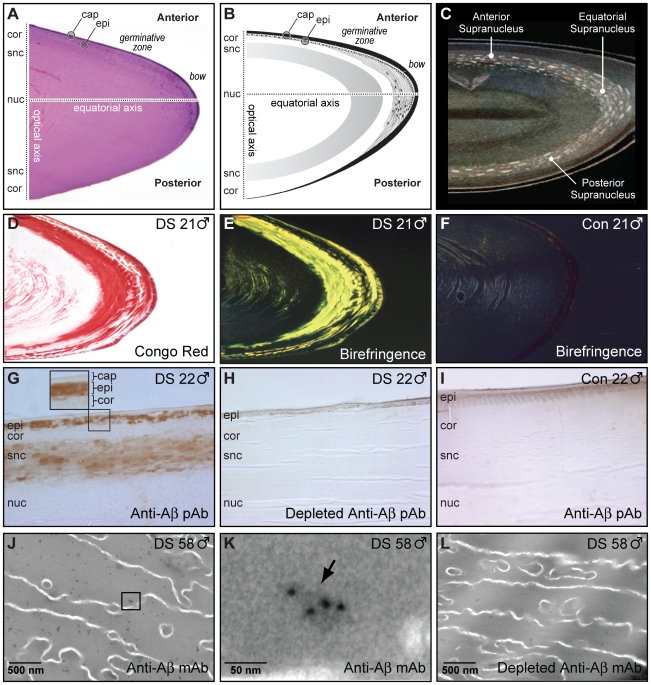
Alzheimer's disease amyloid-β (Aβ) pathology in Down syndrome lens. (**A**) Histological section of a human lens obtained from a 21-year-old male subject with Down syndrome. Hematoxylin and eosin. (**B**) Schematic diagram identifying anatomical regions of the human lens (adapted from *Histology of the Human Eye: An Atlas and Textbook* [52]). (**C**) Archival rendering of classical coronary cerulean “flake” arcuate cataracts in a 40-year-old male with presumptive Down syndrome (from Lowe, 1949 [23]). This historical folio drawing illustrates the mature Down syndrome supranuclear cataract phenotype with dominant subequatorial localization and anteroposterior extension. (**D-F**) Congo red amyloid histochemical analysis of lenses from a 21-year-old male with Down syndrome and age-matched normal male control. (**D**) Congophilia in the cortex and supranuclear subregion of the lens from a 21-year-old male with Down syndrome. (**E**) Intense co-localizing apple-green birefringence in the corresponding cortical and supranuclear subregions of the same Congo red-stained Down syndrome lens imaged with cross-polarized illumination. (**F**) Amyloid histochemical analysis of a lens from a 21-year-old normal control subject did not demonstrate Congophilia nor classical apple-green birefringence under identical cross-polarized illumination. (**G**) Aβ immunoreactivity in the epithelium, deep cortex, and supranuclear regions in a lens from a 22-year-old male with Down syndrome. Inset, magnified detail of the anterior lens (box). *Cap*, capsule; *epi*, epithelium; *cor*, cortex; *snc*, supranucleus; *nuc*, nucleus. (**H**) Confirmation of anti-Aβ antibody specificity in the same Down syndrome lens by immunodepletion of the detection antibody with synthetic human Aβ. Adjacent section of the same Down syndrome lens in Fig. 3G (**I**) Absence of Aβ immunoreactivity in the lens of a normal 22-year-old male control subject. (**J**) Anti-Aβ immunogold electron microscopic analysis of lens from a 58-year-old male with Down syndrome. Heterogeneously distributed anti-Aβ immunoreactive protein aggregates of dimensions ∼5–200 nm localize heterogeneously within the lens fiber cell cytoplasm. Aβ immunoreactivity was not detected at the plasmalemma. Boxed region denotes magnified area shown in Fig. 3*k*. Scale bar = 500 nm. (**K**) High-magnification electron micrograph of a single Aβ-immunoreactive cytoplasmic protein aggregate (arrow) in a lens fiber cell from same Down syndrome lens section shown in Fig. 3*J.* Multiple immunogold particles detect a single cytoplasmic protein aggregate with longest axial cross-section ∼50 nm. Scale bar = 50 nm. (**L**) Confirmation of anti-Aβ antibody specificity. Anti-Aβ immunostaining was not detected in Down syndrome lens when probed with immunodepleted anti-Aβ antibody.

The phenotypic correspondence of the distinctive cataracts in DS and AD encouraged us to investigate the possibility of Aβ pathology in DS lenses. Orientation to the anatomy of the adult human lens is shown (Fig. *3A,B*) and compared to an historical drawing (Fig. *3C*) illustrating the distinctive localized supranuclear lens pathology in a 37-year-old subject with clinical features consistent with Down syndrome [23]. Amyloid histochemical analysis of DS lenses in the present study demonstrated Congo red staining (Fig. 3*D*) and intense apple-green birefringence (Fig. *3E*) under cross-polarized illumination, findings consistent with the tinctorial requirements for designation as amyloid pathology [30], [31]. Amyloid histopathology was not evident in a lens from an age- and sex-matched normal control donor (Fig. 3*F*) and co-localized with lens pathology and Aβ immunoreactivity as demonstrated by specific Aβ immunoreactivity in the deep cortical, supranuclear, and anterior epithelial subregions (Fig. 3*G*). Anti-Aβ immunoreactivity was not detected in adjacent control sections probed with immunodepleted detection antibody (Fig. 3*H*) or in lens specimens from age- and sex-matched normal control donors (Fig. 3*I*). Anti-Aβ immunogold electron microscopy revealed immunoreactive aggregates (∼5–50 nm) that localized to the cytoplasm of supranuclear fiber cells in DS lenses (Fig. 3*J,K*) but was not observed in adjacent control sections probed with immunodepleted detection antibody (Fig. 3*L*). These histopathological findings are identical to those previously described for lens pathology in late-onset sporadic AD [28].

### Identification and Biochemical Characterization of Aβ in Down Syndrome Lens

**Figure 4:**
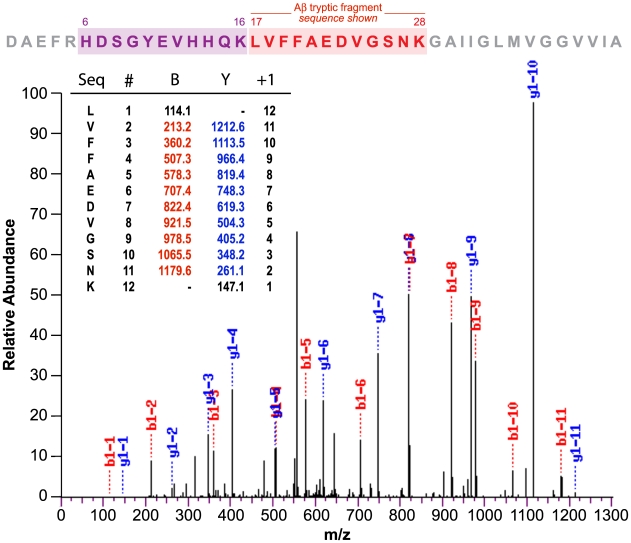
Peptide sequencing of human Aβ from Down syndrome lens. Tryptic digest tandem mass spectrometry sequencing of a ∼4 kDa HPLC eluate derived from human Down syndrome lens protein extract. The retention time of the immunopurified HPLC eluate used for sequencing was identical to synthetic human Aβ. The red shade box denotes the detected 12-residue internal tryptic peptide sequence _17_LVFFAEDVGSNK_28_ that uniquely identifies human Aβ. We detected a second unique tryptic peptide _6_HDSGYEVHHQK_16_ in another analysis (purple underline).

**Figure 5:**
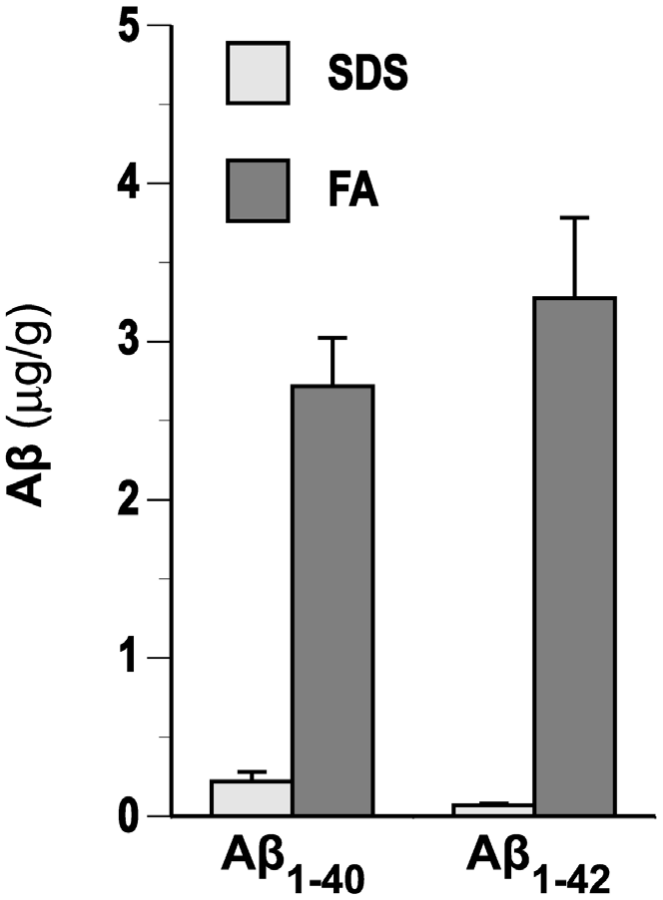
Lens Aβ extraction efficiency in sodium dodecyl sulfate and formic acid. See text for details.

**Figure 6:**
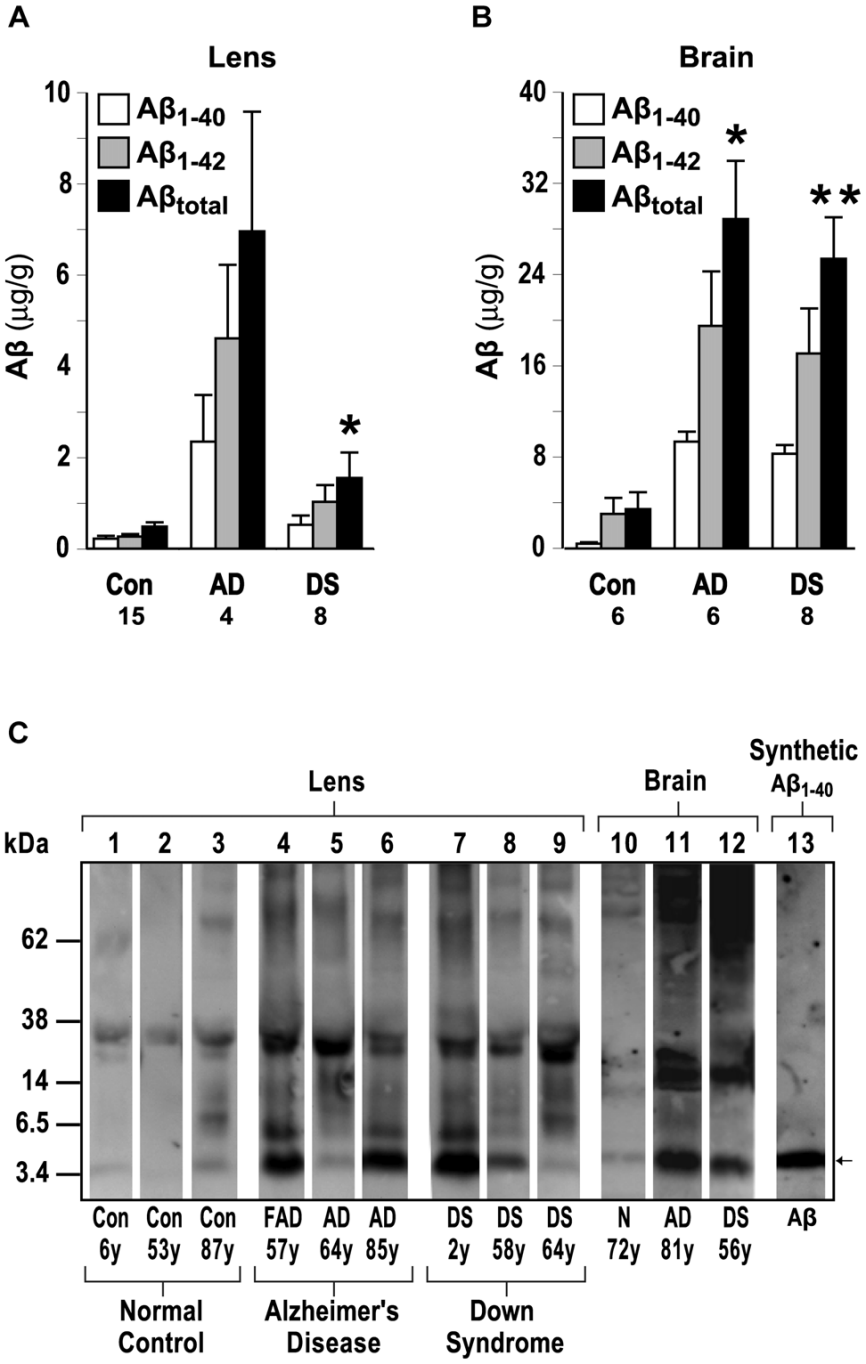
Increased Aβ expression in Down syndrome lens and brain. (**A-D**) Anti-Aβ ELISA analysis of human lens (**A**) and brain (**B**) fractionated by Aβ isoform. (**A**) Lens homogenates demonstrate elevated lens Aβ in subjects with Down syndrome (n = 8; p = 0.027) compared to normal controls (n = 15). (**B**) Brain homogenates demonstrate elevated Aβ in subjects with Down syndrome (n = 8; p = 0.007) and Alzheimer's disease (n = 6; p = 0.026) compared to normal controls (n = 6). (**C**) Immunoblot analysis of Down syndrome lens and brain homogenates reveals an intense Aβ-immunoreactive band that migrated with an apparent molecular weight of ∼4kDa corresponding to synthetic human Aβ monomer (arrow). Note strong band corresponding to Aβ monomer in the 2-year-old Down syndrome lens (lane 7) and apparent shift from lower- to higher-order oligomeric Aβ in Down syndrome lenses with advancing age (lanes 7–9). Protein loading was normalized in each lane.

Definitive identification of human Aβ in DS lens was accomplished by tryptic digest tandem mass spectrometry peptide sequencing. Biochemical sequencing analysis was conducted on small molecular weight eluates derived from HPLC fractionation of human DS lens protein extract (Fig. 4). In positive electrospray mode, the LC-MS/MS spectrum detected two tryptic peptide signals corresponding to _17_LVFFAEDVGSNK_28_ and _6_HDSGYEVHHQK_16_ that uniquely identify human Aβ. The detected fragments did not derive from the larger amyloid precursor protein (APP) as sequencing was performed on eluates with retention times corresponding to monomeric Aβ (∼4 kDa). In order to establish the tissue concentration of Aβ in the lens, we first investigated the relative efficiency of sodium dodecyl sulfate compared to formic acid extraction performed on protein extracts obtained from human lens homogenates (Fig. 5). Formic acid demonstrated a far superior extraction profile relative to sodium dodecyl sulfate for both Aβ1-40 and Aβ1-42. ELISA results (Fig. 6*A,B*) demonstrated elevated lens and brain tissue levels of the major human Aβ isoforms (Aβ_40_ and Aβ_42_) and total Aβ that were consistently elevated in DS compared to normal controls and comparable to the values previously reported in AD [28]. Immunoblot analysis of DS lens extract (Fig. 6*C*) revealed a prominent Aβ-immunoreactive band that migrated with an apparent molecular weight of ∼4 kDa corresponding to monomeric Aβ that was also detected in homogenate of AD brain. The apparent Aβ monomer bands detected in DS and AD lens and brain homogenate migrated with the same electrophoretic mobility observed in the single band detected in a sample of purified synthetic human Aβ40. Aβ monomer and apparent oligomeric species were also detected as weak but distinct bands in normal control lens. Total Aβ immunoreactive material detected in the lens of a 2-year-old DS subject was comparable to that detected in lenses from a 57-year-old subject with familial AD and an 85-year-old subject with neuropathologically-confirmed late-onset AD (Fig. 6*C*). The results of the immunoblot analysis provide confirmatory evidence supporting the finding of Aβ in DS lenses and also demonstrate an apparent increase in oligomeric Aβ and corresponding decrease in monomeric Aβ in the lens as a function of advancing age.

### Down Syndrome Aβ Lens Pathology Recapitulated in a Model Lens Protein System *In Vitro*

**Figure 7:**
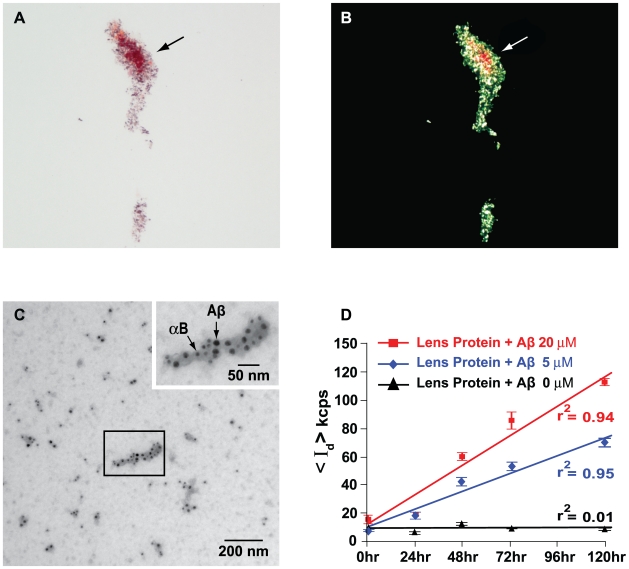
Aβ potentiates lens protein aggregation and Rayleigh light scattering *in vitro.* (**A**) Brightfield photomicroscopic image of a Congo red-stained protein aggregate (arrow) formed during incubation of human lens protein extract with synthetic human Aβ. (**B**) Same Congo red-stained lens protein aggregate demonstrating classical amyloid birefringence under cross-polarized illumination. (**C**) Anti-Aβ/anti-αB-crystallin double immunogold electron microscopic analysis of protein aggregates formed during incubation of human lens protein with synthetic human Aβ. Larger immunogold particles (15 nm diameter) detect Aβ. Smaller immunogold particles (10 nm diameter) detect αB-crystallin. Box indicates region of detail highlighted in Fig. 7*D*. Scale bar = 200 nm. Inset, hetero-oligomeric composition noted in a single protein aggregate demonstrated by double immunogold (anti-Aβ and anti-αB-crystallin) electron microscopy. Analysis was performed on human lens protein incubated with synthetic human Aβ. Larger immunogold particles (15 nm diameter) detect Aβ (black arrow). Smaller immunogold particles (10 nm diameter) detect αB-crystallin (white arrow). Scale bar = 50 nm. (**D**) Quasi-elastic light scattering (QLS) analysis detects increased backscattered light due to concentration-and time-dependent protein aggregation during incubation of human lens protein with synthetic human Aβ. Experimental samples studied by QLS were analyzed by amyloid histochemistry (Fig. 7A,B) and double (anti-Aβ and anti-αB-crystallin) immunogold electron microscopy (Fig. 7C).

These data indicate that Aβ accumulates in the cytoplasm of supranuclear lens fiber cells in individuals with DS. We previously showed that Aβ binds (Kapp ∼20 nmol/L) and co-aggregates with αB-crystallin (HspB5) [28], a prototypic molecular chaperone and small heat shock protein [32] that is abundantly present in the lens fiber cell cytoplasm and upregulated in AD brain [33]. We hypothesized that accumulation of amyloidogenic Aβ peptides in the lens fiber cell would potentiate cytosolic lens protein aggregation and increase Rayleigh light scattering. To investigate this hypothesis, we incubated synthetic Aβ with human lens protein extract. Addition of Aβ to human lens protein extract generated birefringent congophilic aggregates consistent with amyloid (Fig. 7*A,B*). Double immunogold electron microscopic analysis revealed electron-dense aggregates that co-immunostained for Aβ and αB-crystallin (Fig. 7*C*). These hetero-oligomeric aggregates demonstrated comparable size, morphology, and immunoreactivity relative to the cytosolic protein aggregates identified in postmortem DS lens specimens. Significantly, the dimensions of these Aβ-containing protein aggregates (∼5pone.0010645 pone.0010646 200 nm in longest axial cross-section) describe classical Rayleigh light scattering centers. Therefore, we sought to determine whether Aβ potentiated lens protein aggregation as assessed by quasi-elastic light scattering (QLS) analysis. Addition of synthetic human Aβ to human lens protein extract resulted in dose- and time-dependent increases in backscatterd light intensity that was not observed in control lens protein extracts without added human Aβ (Fig. 7*D*). These findings are consistent with Aβ-potentiated lens protein aggregation.

## Discussion

In this report, we identify the origin and pathogenic mechanism of cataractogenesis in DS, establish the distinctive lens phenotype in DS as a genetic cataract, and define the molecular correspondence of DS-associated pathology in the lens and brain. Taken together with our previous findings of supranuclear Aβ lens pathology in late-onset sporadic AD [28], these results also establish the pathogenic relationship linking Aβ pathology in the lens and brain in both DS and AD.

AD neuropathology is an invariant feature of DS in subjects over the age of 30 [18], [19] but may emerge as early as the first decade [18], [34]. A critical factor contributing to the association of DS and AD Aβ pathology is the locus of the amyloid precursor protein gene (*APP,* 21q21) on the long arm of chromosome 21 [16], [35], [36]. Chromosome 21 triplication leads to increased dosage of the *APP* gene, accelerated cerebral Aβ accumulation, progressive AD neuropathology, and age-dependent cognitive sequelae [18], [37], [38], [39], [40]. With the exception of a single reported case of an individual with an atypical DS karyotype involving a specific chromosome 21 micro-deletion in the *APP* gene locus [41], all subjects with Down syndrome (including the rare cases involving Robertsonian translocation, partial duplications, or trisomic mosaicism) demonstrate triplication of the *APP* gene locus (21q21), overexpress amyloid precursor protein (APP) in affected somatic cells, accumulate β-amyloid peptides (Aβ) in the brain, and reveal evidence of early-onset Alzheimer's disease (AD) neuropathology [18], [19], [34], [40], [42].

**Figure 8:**
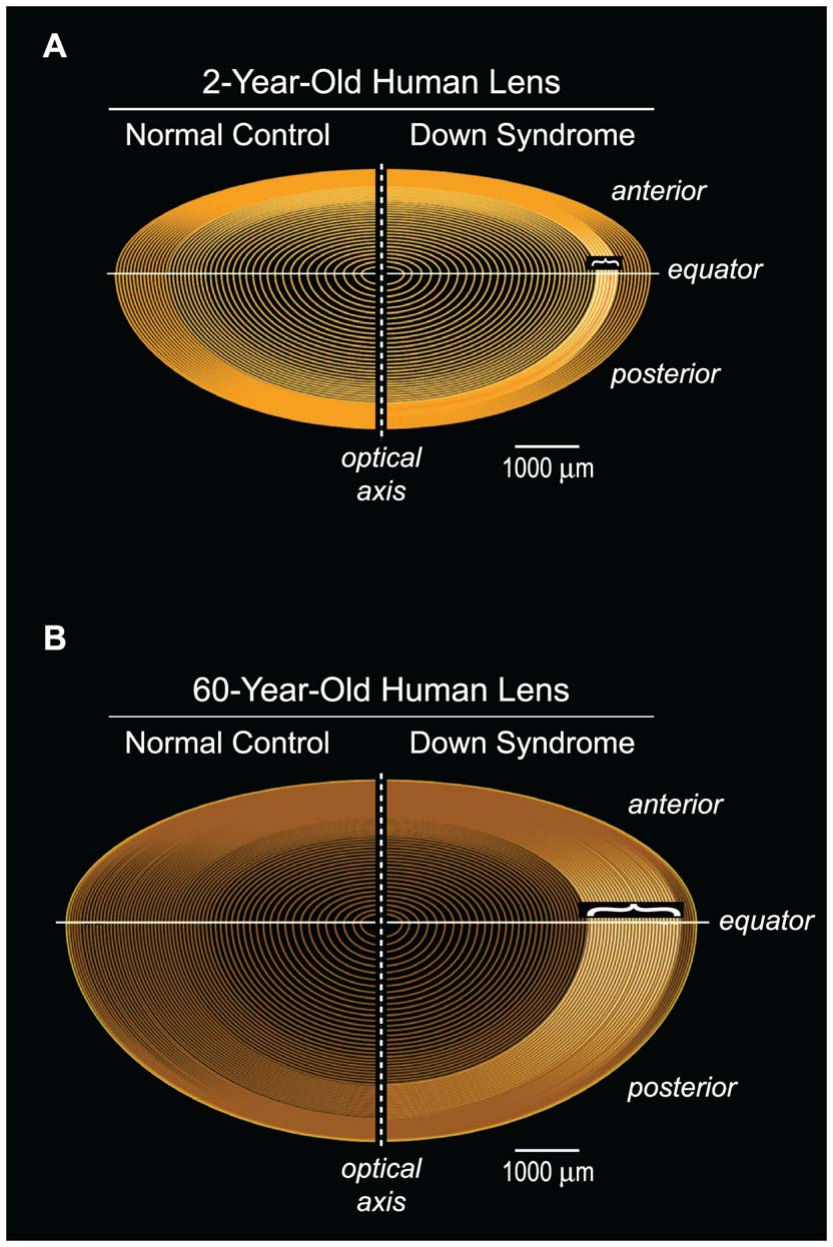
Age-dependent supranuclear cataractogenesis in Down syndrome. The anatomical localization of the characteristic Down syndrome cataract phenotype (white shading) reflects the temporal origin and natural history of the underlying lens pathology. Fetal lens fiber cells are not involved in this pathogenic process. Parentheses indicate equatorial axial extent of age-dependent disease-linked Aβpathology in the supranuclear subregion of Down syndrome lenses. See text for details.

In this report, we hypothesize and confirm that this genotype-phenotype concordance extends to Aβ-linked molecular pathology in the supranucleus and deep cortex of the lens. The characteristic cataract phenotype associated with Aβ lens pathology has been identified in only two clinical disorders, Alzheimer's disease [28] and as reported here, Down syndrome. This distinctive supranuclear cataract phenotype has not been reported in normal individuals, and is not observed in other non-AD neurodegenerative diseases, nor in normal aged controls [28]. The classical cerulean “blue dot” cataracts characteristic of DS initially emerge at the equatorial periphery of the lens, and over time, encompass progressively larger areas of the supranucleus and deep cortex (Fig. 8). While lens pathology observed in both infantile and mature DS lenses reveal opaque regions that are clinically described as supranuclear, the ages of the corresponding fiber cells comprising these regions are different (Fig 8). In this respect, it is important to note that the lens pathology in DS lenses does not implicate involvement of lens fibers cells in the fetal nucleus.

Distinctive lens pathology is recognized as a characteristic early-onset ocular phenotype in subjects with DS that may be clinically detectable early in life, and in some cases, at birth [20], [22], [23], [24], [26], [43], [44]. By contrast, DS neuropathology and cognitive sequelae typically emerge in the third decade of life and age-dependently progress thereafter [18], [19], [37], [38], [39], [40]. In the present study, early-onset lens pathology is histochemically documented in illustrative cases of two young adults (21 and 22 years of age) with DS. Lenses obtained from these donors revealed marked Aβ amyloid lens pathology that was evident not only in the supranuclear subregion, but also throughout the anterior and equatorial cortex and epithelium. Aβ biochemical analysis of DS and normal control lenses indicates that increased Aβ expression in the lenses of people with DS may be evident very early in life. In one case included in this study, we detected abnormally high tissue levels of Aβ in the lens of a DS subject at 2 years of age. Although visual impairment may not become clinically apparent due to the peripheral distribution of these lenticular lesions relative to the iris, it is certainly the case that Aβ molecular pathology in the lens may be amongst the earliest expressed age-dependent phenotypes in Down syndrome.

The DS-associated cataract phenotype is not observed in other non-AD neurodegenerative diseases, nor in normal aged controls. In this context, it is important to note that DS-associated cataracts are phenotypically distinct from age-related cataracts (ARC) that commonly emerge starting in the fifth decade of life and typically localize in the central nuclear region. In contrast to the lens pathology in ARC, the DS lens phenotype is often clinically detectable early in life, and in some cases, may be present at birth [20], [22], [23], [24], [26], [43], [44]. The distinctive lens phenotype associated with DS is further distinguished from ARC by supranuclear Aβ histopathology, a pathological feature shared with late-onset AD [28].

**Figure 9:**
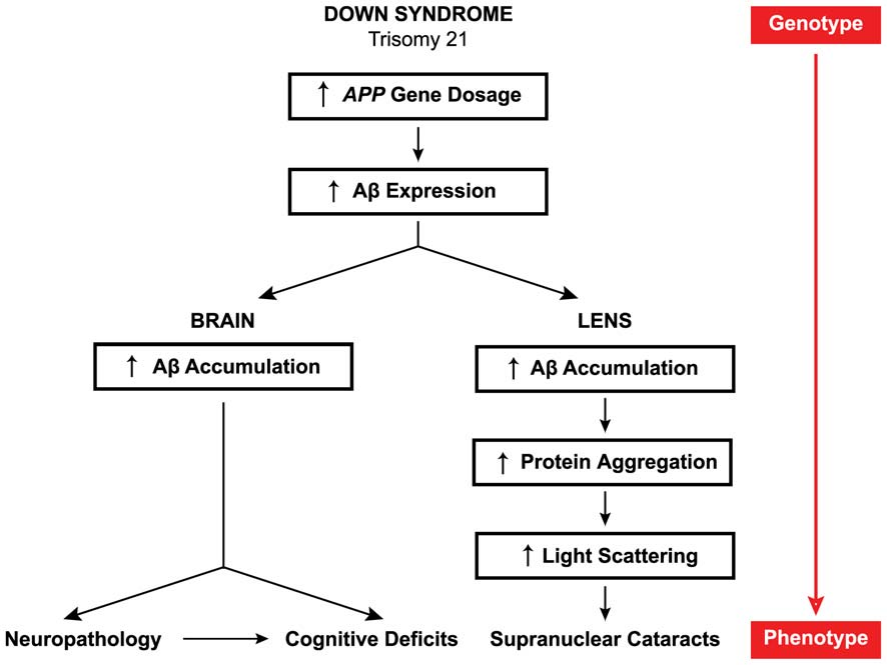
Model pathogenic pathways in Down syndrome brain and lens. Triplication of human chromosome 21 in Down syndrome results in increased dosage of the *APP* gene (21q21), overexpression of the Alzheimer's disease amyloid-β precursor protein (APP), and progressive accumulation of amyloidogenic amyloid-β peptides (Aβ) in the brain and lens. Deposition of Aβ in both anatomical compartments results in age-dependent Aβ amyloid pathology and disease-linked tissue-specific phenotypes in both Down syndrome and Alzheimer's disease. See text for details.

The results of the present study support a DS-associated pathogenic pathway linking progressive age-dependent Aβ accumulation in the lens and supranuclear cataractogenesis with corresponding cerebral Aβ accumulation and neuropathology in the brain. We propose the following mechanistic pathway leading to expression of the characteristic lens and brain phenotypes associated with DS (Fig. 9). Increased *APP* gene dosage resulting from chromosome 21 triplication (*APP,* 21q21) leads to increased expression of the amyloid precursor protein (APP) and elevated levels of the pathogenic Aβ cleavage peptides in affected tissues. In the brain, Aβ accumulation leads to early-onset neuropathology and progressive age-dependent neurocognitive sequelae. With respect to the lens, the present investigation supports the hypothesis that increased expression of Aβ in the lens leads to amyloidogenic interactions with other structural proteins (e.g., αB-crystallin) within the cytoplasm of supranuclear lens fiber cells. This pathogenic cascade leads to increased lens protein aggregation, light scattering, opacification, and ultimately, disease-linked cataracts [45]. Given the fact that the long-lived lens fiber cells are metabolically sluggish and demonstrate limited capacity to catabolize aggregated protein, we speculate that DS-linked Aβ amyloid pathology in the lens may precede corresponding Aβ pathology in the brain. Regardless, the proposed pathogenic model for Aβ pathology in both tissue compartments (brain and lens) is in general agreement with a broad conception of the amyloid cascade hypothesis [16], [46], [47].

We hypothesize that the Aβ detected in DS lenses is generated endogenously. The human lens expresses the full complement of enzymes and precursor proteins necessary to generate and process Aβ [28], [48], [49], [50]. In the present study, this hypothesis is supported by the finding of intense Aβ immunoreactivity in DS lens epithelial cells. Our finding that lens fiber cell Aβ is exclusively cytoplasmic suggests that this amyloidogenic peptide may be released from intracellular processing compartments during organelle disintegration accompanying epithelial-to-fiber cell terminal differentiation. This model points to the lens as an appropriate target for ascertaining the temporal origin and natural history of AD-linked molecular pathology *in vivo.*

Our findings suggest an apparent temporal discordance in DS with respect to phenotype expression in the lens and brain, with pathology in the former preceding that in the latter. However, we also observed variability in age-dependent phenotypic expression of Aβ molecular pathology in the lens that comports with analogous variability in the brain of subjects with DS [34], [51]. The significance and natural history of variability in Aβ tissue concentration in the lens and its possible relationship to the brain in DS are subjects of ongoing investigation.

In this study, we identify Aβ amyloid pathology as the shared molecular etiology of two defining features of DS, namely, supranuclear cataracts in the lens and AD neuropathology in the brain. The proximal pathogenesis of both pathologies likely derives from the primary chromosomal disorder and associated *APP* gene-dosage imbalance (Fig. 9). The results reported here support a testable pathogenic mechanism by which the primary chromosomal disorder in DS may be translated into a distinctive phenotype expressed in the lens. In this regard, the phenotypic concordance of Aβ lens pathology in both diseases, DS and AD, is striking. Given that DS-linked pathology may be indicative of aberrant Aβ accumulation in both compartments, the lens may provide an optically accessible target tissue for early detection and longitudinal assessment of early Aβ molecular pathology in DS and AD. Future clinical studies will be required to systematically evaluate the frequency of this supranuclear phenotype in DS patients. Quasi-elastic light scattering instrumentation or other techniques may provide a practical approach for quantitative assessment of specific lens pathology throughout the course of the disease.

## Supporting Information

Figure S1 Stereo image pair demonstrating mature supranuclear pathology in the lens (with intact zonule fibers) from a subject with Down syndrome. Characteristic circumferential supranuclear cataract in the lens of a 64-year-old male subject with Down syndrome. This distinctive cataract is evident as an annular half-toroid band of opacification in the deep cortical and supranuclear subregions of the lens (shown with intact zonules). This same lens specimen is presented as a slit lamp image (Fig. 1H) and as a stereo image pair (without zonules, Fig. 2A). This dramatic Down syndrome cataract is phenotypically comparable to the subequatorial supranuclear cataract observed in advanced Alzheimer's disease. These distinctive supranuclear cataracts are not observed in age-normal control subjects. See text for details.
(2.77 MB TIF)
