# Supplementary figures and images for: Alzheimer's Disease Amyloid-β Links Lens and Brain Pathology in Down Syndrome
Source: PLoS One. 2010 May 20;5(5):e10659. doi: 10.1371/journal.pone.0010659 (PMC2873949; doi:10.1371/journal.pone.0010659)

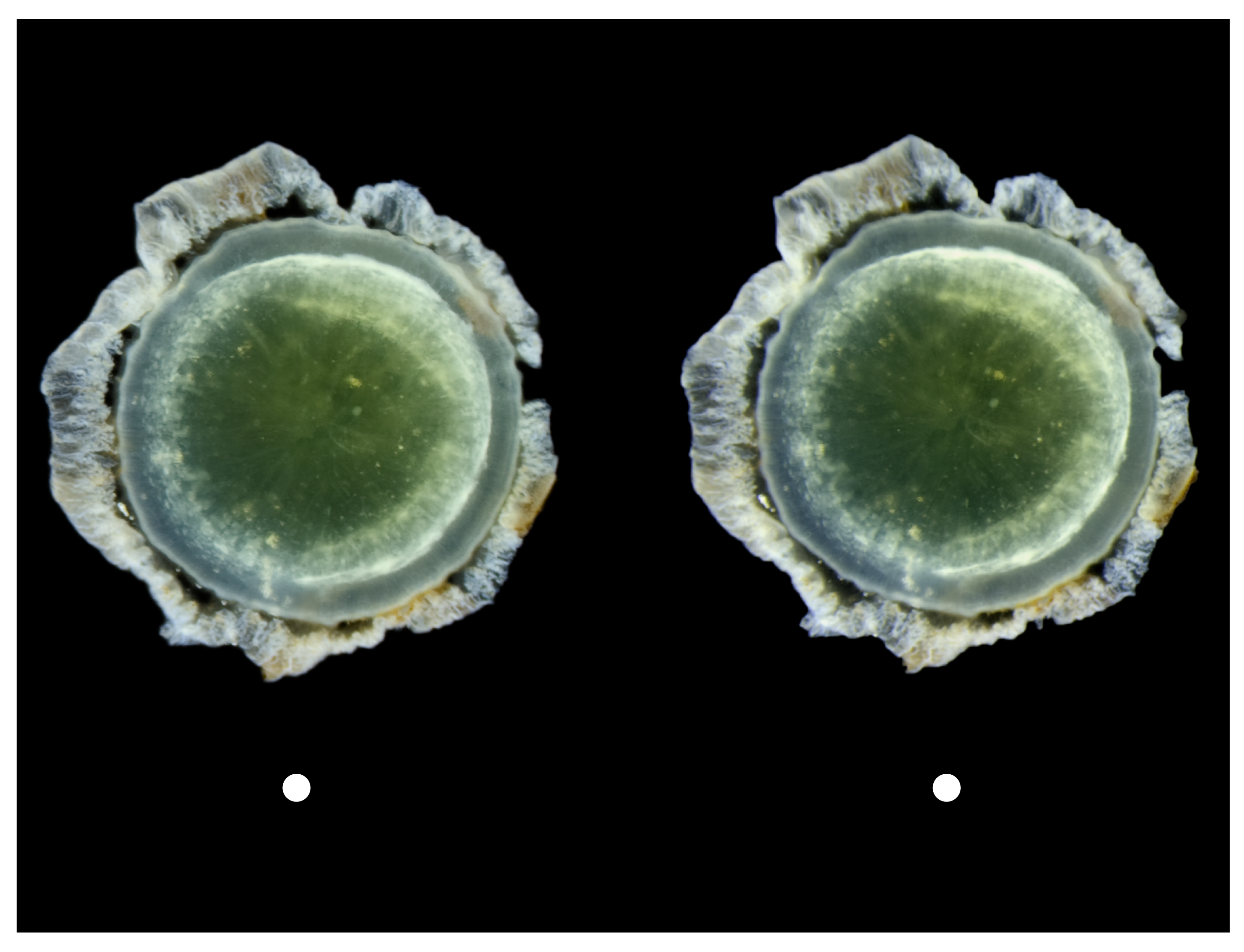

Supplement: Figure S1 — Stereo image pair demonstrating mature supranuclear pathology in the lens (with intact zonule fibers) from a subject with Down syndrome. Characteristic circumferential supranuclear cataract in the lens of a 64-year-old male subject with Down syndrome. This distinctive cataract is evident as an annular half-toroid band of opacification in the deep cortical and supranuclear subregions of the lens (shown with intact zonules). This same lens specimen is presented as a slit lamp image (Fig. 1H) and as a stereo image pair (without zonules, Fig. 2A). This dramatic Down syndrome cataract is phenotypically comparable to the subequatorial supranuclear cataract observed in advanced Alzheimer's disease. These distinctive supranuclear cataracts are not observed in age-normal control subjects. See text for details. (2.77 MB TIF) [file pone.0010659.s001.tif]
